# Semaphorin-4D (Sema-4D), the Plexin-B1 ligand, is involved in mouse ovary follicular development

**DOI:** 10.1186/1477-7827-5-12

**Published:** 2007-03-21

**Authors:** Avital Regev, Shlomit Goldman, Eliezer Shalev

**Affiliations:** 1 Laboratory for Research in Reproductive Sciences, Department of Obstetrics and Gynecology, Ha'Emek Medical Center, 18101, Afula, Israel; 2 Rappaport Faculty of Medicine, Technion-Israel Institute of Technology, Haifa, Israel

## Abstract

**Background:**

Human Plexin-B1 is expressed in two truncated forms. The long form encodes a trans-membranal protein, while the short form, which is bound to the cell surface and partially secreted, possibly serves as a decoy receptor. Plexin receptors are trans-membrane proteins. The sema domain, found in the extracellular region, is common to all plexins, semaphorins, and the scatter factor receptors and is crucial for the biological activity and plexin receptor specificity. Semaphorin-4D/Plexin-B1 binding provides attractive and repulsive cues for the navigation of axonal growth cones, and new studies suggest that this system also plays a role in the regulation of the biological functions of endothelial cells, specifically in the control of angiogenesis. In a previous study, we have demonstrated the expression and possible role of Plexin-B1 in the mouse ovary. The present study was designed to test the hypothesis that Plexin-B1 effects are mediated by Semaphorin-4D.

**Methods:**

In vivo expression and localization of mouse ovarian Sema-4D were tested by immunohisto-chemistry. The role of Sema-4D in follicular development was examined by in vitro growth of preantral follicles in the presence or absence of Semaphorin-4D, with or without neutralizing antibodies against Plexin-B1. Follicular growth and steroid hormone secretion rates were tested.

**Results:**

Semaphorin-4D is expressed in the mouse ovary in vivo mostly in the granulosa cells and and its expression is modulated by PMSG and hCG. In the presence of Semaphorin-4D, in-vitro constant growth was observed as indicated by follicular diameter during the culture period and elevated steroid hormone secretion rates compared with control. These effects were abolished after addition of neutralizing antibodies against Plexin-B1.

**Conclusion:**

In the ovarian follicle, the effect of Plexin-B1 is mediated by sema-4D.

## Background

Semaphorins and Plexins are independently identified protein families that share a striking structural similarity and are believed to derive from a common ancestor [1]. Semaphorins were originally characterized in the nervous system, where they have been implicated in repulsive axon guidance [2].

The class 4 Semaphorin (Sema-4D), through binding of its receptor, Plexin-B1, exerts important biological effects on a variety of cells. A significant role for these molecules has been established in cardiac and skeletal development [3], immune response [4], epithelial morphogenesis [5] and tumor growth and metastasis [6].

Plexin-B1 is highly expressed in endothelial cells (EC) and its activation by Sema-4D elicits a potent pro-angiogenic response and induces EC migration [7]. Sema-4D/Plexin-B1 binding sustains proliferation and survival of normal and leukemic CD5+ B lymphocytes [8].

Plexin activation hinders cell attachment to adhesive substrates, blocks the extension of lamellipodia, and thereby inhibits cell migration [9].

Plexin activation in adhering cells rapidly leads to retraction of cellular processes and cell rounding (cell collapse) [10].

Sema-4D is able to activate the invasive growth program in epithelial cells, a complex process including dissociation of cell-to-cell adhesive contacts, anchorage-independent growth, and branching morphogenesis [5].

The plexin-semaphorin system is implicated in cyto-skeletal re-organization, adhesion and cell proliferation. All these functions are utilized during the process of follicular development during the estrus cycle. The growing follicle must reshape by creating an antrum and at least two cell populations. The follicle also must expand and proliferate in order to grow into mature graafian follicle. We hypothesize that the effect of Plexin-B1 in the ovarian follicle is mediated by sema-4D. The present study was designed to test this hypothesis.

## Methods

### Animals

All experiments were conducted using female ICR mice (Harlan, Israel), housed and bred in a temperature- and light-controlled room, and provided food and water. The study was approved by the local ethics committee (840201).

**(a) **– Thirty female mice, 26 days old, were injected with 5 IU pregnant mare serum gonadotropin (PMSG). Ten mice were sacrificed 48 hours following injection. The remaining 20 mice were injected with 5 IU human chorionic gonadotropin (hCG), 24 hours after PMSG injection. Ten were sacrificed 6 hours following injection and the last 10 were sacrificed 24 hours following injection. A group of 10 immature female mice that were not treated served as control for untreated mice. The ovaries were fixed in 4% formalin and embedded in paraffin for immunohistochemistry analysis

**(b) **– Preantral follicles were collected from sixteen, 12-day-old, female ICR mice by ovarian micro-dissection.

### Isolation of preantral follicles

Ovaries from 12-day old ICR mice were removed aseptically, separated from the connective tissue, and placed in 2.5 mL of M-199 supplemented with 5% fetal calf serum (FCS), 100 mIU/mL penicillin, and 100 mg/mL streptomycin (all from Biological Industries, Beit-Ha'Emek, Israel) at 37°C. For each mouse, ovaries were dissected mechanically using a 26-gauge needle in M-199 medium supplemented with 5% FCS.

### Culture of preantral follicles

Isolated follicles were rinsed three times in 30 μL of culture medium composed of M-199 medium supplemented with 100 mIU/mL FSH and 100 mIU/mL LH (Pergonal, Serono laboratories), 5% FCS [11] in the absence or presence of Sema-4D (50 ng/ml) kindly donated by L. Tamagnone [5] (Institute for Cancer Research and Treatment, University of Turin Medical School, Turin, Italy) with and without 200 ng/mL neutralizing antibodies against Plexin-B1 (Santa Cruz Biotechnology, Inc., Santa Cruz, CA). An intact preantral follicle was defined as a round follicular structure containing a visible oocyte centrally located and surrounded by more than one granulosa cell layer (generally two layers) with at least one completed granulosa cell layer. Follicles were measured under 100× magnification with a pre-calibrated ocular micrometer. A total of 151 Follicles of 100–130 μm were selected and individually cultured for 12 days in 24-well Falcon culture dishes (Becton Dickinson, Le Pont de Claix, France) using 35 μL of culture medium under 0.6 mL of mineral oil (Sigma Aldrich, St. Louis, MO) at 37°C in a humidified atmosphere of 5% CO_2 _in air. On days 3, 4, 5, 8, 9, 10, 11 and 12 of the culture period, 10 μL of culture medium from each drop was collected and frozen for subsequent hormonal assay. After 9 days of culture, 1.5 IU/mL Human chorionic gonadotropin (hCG) (Ares Serono, Geneva, Switzerland) was added to the culture medium to induce follicular rupture [12].

### Hormonal measurements

In five independent experiments, conditioned media from surviving follicles were collected at different times of culture. Surviving follicles were defined as the attached follicles that had retained their oocytes in the granulosa cell mass. Media were frozen and hormonal measurements were performed at the end of each experiment. Estradiol (E_2_), Progesterone (P_4_), and Testosterone (T) were measured using EIA commercial kits (cat. nos. RE-522-31, RE-520-41, and RE-521-51, respectively; Immuno-Biological Laboratories, Inc., Minneapolis, MN) according to the manufacturer's recommendations.

### Immunohistochemistry

All specimens taken from the untreated and PMSG, hCG treated 26-day old mice were fixed in 4% formalin and embedded in paraffin. For immuno-histochemical staining, the endogenous peroxidase in formalin-fixed paraffin-embedded tissue was inhibited by incubating specimens with 0.5% H_2_O_2 _for 10 minutes before incubation with the primary rabbit polyclonal IgG anti- Sema-4D (Santa cruz, sc-20928). The slides were incubated with anti-Sema-4D primary antibodies at a 1:100 dilution, and developed using the streptavidin-biotin system according to the manufacturer's instructions. Sema-4D was identified as brown membrane staining in ovarian sections counterstained with hematoxylin. Tissue used as positive control in this study was obtained from the spinal cord (data not shown). Negative controls were run routinely in parallel by replacing the primary antibody with rabbit IgG antibody. No specific immuno-reactivity was detected in these negative control specimen tissues.

The immunohistochemistry photos were analyzed by Image Pro software that quantifies density per area. At least 3 sections from each ovary were evaluated.

### Statistics

Each independent experiment was repeated at least 3 times. All data are presented as mean ± SEM. Statistical analysis of the data was carried out with one-way analysis of variance (ANOVA). *t*-tests were used to compare between two groups as post ANOVA test; P < 0.05 was considered significant.

## Results

### In vivo Semaphorin-4D expression

**Figure 1:**
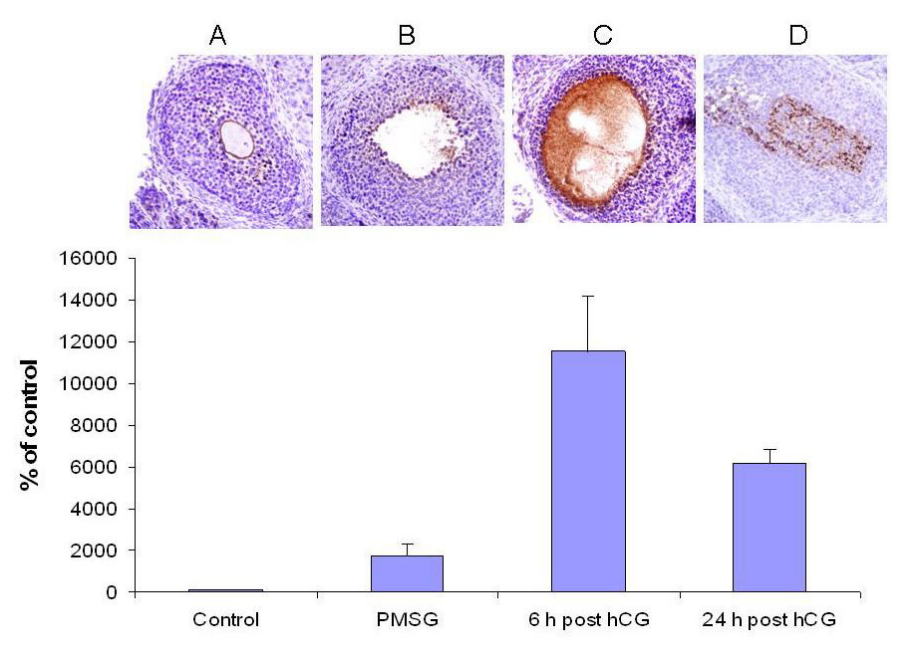
Immunohistochemical staining revealing the presence of Sema-4D in the mouse ovary at all stages: (A) un-stimulated (B) after PMSG administration, (C) 6 hours after hCG administration, and (D) 24 hours after hCG administration. Sema-4D was identified as red-brown membrane staining in ovarian sections counterstained with hematoxylin; magnification ×400. Bar graph describing the relative percentage of Sema-4D expression, representing, mean ± SEM from three independent experiments.

Immunohistochemistry of ovaries obtained from 26-day old untreated and treated mice revealed staining for Sema-4D, mainly in the periluminal granulosa cells, at all stages (Figure 1). Sema-4D staining increased after PMSG or hCG injection (P < 0.0016). Sema 4D was also observed in the follicular lumen six hours following hCG injection. In the control group, representing non-stimulated follicles, low levels of Sema 4D were observed (Figure 1).

### The effect of Sema-4D on in vitro follicular growth and development

In vitro cultures of preantral mouse follicles, obtained from 12-day old ICR female mice, were cultured for 12 days in medium (control, n = 29), medium containing Sema-4D alone (50 ng/ml, n = 34) and medium containing Sema-4D and neutralizing antibodies against Plexin-B1 (200 ng/mL, n = 34). On day 9, hCG was added to the culture. Follicular growth and development were estimated by measuring follicular diameters, using a microscope scale, and represented as mean ± SEM.

**Figure 2:**
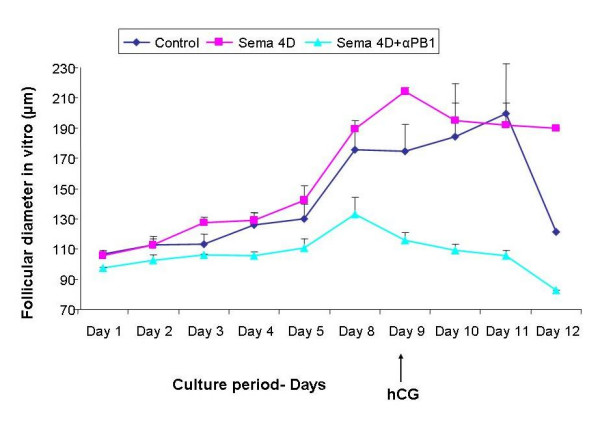
Diameter of follicles obtained from 12-day old ICR mice and grown in in-vitro cultures for 12 days in M-199 Medium containing gonadotropins (FSH and LH 100 mIU/ml) and with the addition of Sema-4D (50 ng/ml) in the presence or absence or of specific neutralizing antibodies against Plexin-B1 (200 ng/ml). On day 9 hCG was added to the culture. Blue diamond represents the follicular diameter in the control group, Pink square represents the follicular diameter in the Sema-4D group. Turquoise triangle represents follicle diameter in the group treated with Sema-4D and neutralizing antibodies against Plexin-B1.

A significant increase in follicular diameter during the culture period was observed in the control group (*P *< 0.05). A statistically significant difference was found between follicular diameter on day 1 of the culture period (mean primary follicular diameter) and follicular diameter on day 11 of the culture period (mean maximal follicular diameter). The mean diameter was 100.65 ± 0.28 versus 190.93 ± 3.32, respectively (P < 0.05; Figure 2).

A significant increase in follicular diameter during the culture period was also observed in the Sema-4D treated group (*P *< 0.05; Figure 2). Comparison of the mean follicular diameter, between day 1 (mean primary follicular diameter) and day 9 of the culture period (mean maximal follicular diameter) in the control group revealed a significant difference between the two groups (the mean diameter was 100.55 ± 0.24 versus 210.42 ± 0.24, respectively, P < 0.05). Comparison of the maximal follicular diameter between the control and Sema-4D treated group revealed no significant difference, (190.93 μm ± 3.32 and 210.42 μm ± 0.24 respectively; Figure 2). However, the period of time needed to reach this diameter in the Sema-4D treated group was significantly shorter compared with the control group (day 9 ± 0.28 versus day 11 ± 0.0001: *P *< 0.03; Figure 2).

The addition of neutralizing antibodies against Plexin-B1 to the follicles treated with sema-4D abolished the effect of sema-4D on follicular growth (*P *< 0.005; Figure 2). Comparison of the mean follicular diameter, between day 1 (mean primary follicular diameter) and day 8 (mean maximal follicular diameter) in the control group, revealed no significant differences (Day 1 90.73 ± 0.06 versus Day 8 130.27 ± 1.13).

However, maximal follicular diameter in the Sema-4D group (day 9 of culture period) was significantly higher than in the group treated with Sema-4D and neutralizing antibodies against Plexin-B1 (day 8 of culture period) (210.42 μm ± 0.24 and 130.27 μm ± 1.13, respectively, *p *< 0.005; Figure 2)

### The effect of Sema-4D on steroidogenesis

Conditioned media from control follicles (n = 18), follicles treated with Sema-4D alone (50 ng/ml, n = 18), and follicles treated with Sema-4D and neutralizing antibodies against Plexin-B1 (50 ng/ml and 200 ng/mL, respectively, n = 18) were analyzed for E_2_, P_4 _and T secretion on days 3, 4, 5, 8, 9, 10, 11 and 12 of the culture period.

**Figure 3:**
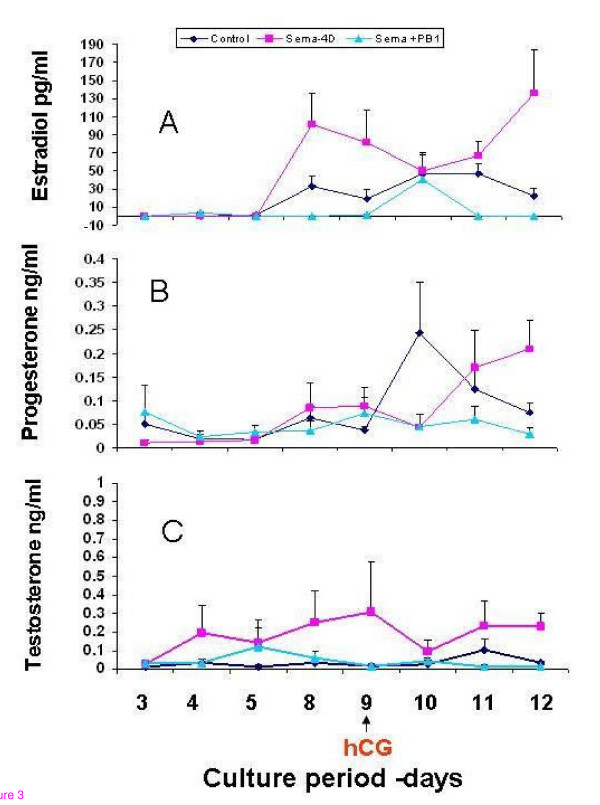
Secretion of steroid hormones (A. Estradiol; B. Progesterone and C. Testosterone), obtained from conditioned media from cultured follicles over 12 days in the absence (Dark blue circles) or presence of Sema-4D (Pink square) or in combination with specific neutralizing antibodies against Plexin-B1 (turquoise triangles). The graph represents mean ± SEM of the steroid hormones secreted from three independent experiments in triplicate.

As illustrated in Figure 3A, the E_2 _secretion rate was significantly increased during the culture period in the control and Sema-4D groups (*P *< 0.0026 and *p *< 0.001, respectively). Comparison of E_2 _levels in each group during the culture period revealed a significant difference (mean peak E_2 _level in the control 46.92 ± 11.49 versus 101.41 ± 34.60 in the sema-4D group, *P *< 0.001). The addition of neutralizing antibodies against Plexin-B1 (200 ng/mL) abolished the increase seen with Sema-4D (mean peak E_2 _levels in the sema-4D group 101.41 ± 34.6 versus 41.06 ± 12.8 pg/ml in the presence of neutralizing antibodies against Plexin-B1; *P *< 0.001).

Figure 3B illustrates the P_4 _secretion rate in the control and study groups. In the control and Sema-4D groups, P_4 _secretion rate remained unchanged during the first days of the culture period and increased significantly on days 10 and 12, respectively (*P *< 0.005.) Comparison between the mean peak of P_4 _secretion level in the control versus sema-4D group, revealed no significant change (0.24 ± 0.1 versus 0.17 ± 0.08, respectively). In the presence of neutralizing antibodies against Plexin-B1 (200 ng/mL), the P_4 _secretion rate remained unchanged during the culture period.

Comparing the peak P_4 _levels between follicles treated with Sema-4D and follicles treated with neutralizing antibodies against Plexin-B1 revealed a significant difference between the two groups (0.17 ± 0.08 versus 0.07 ± 0.03 ng/ml progesterone secretion; *P *< 0.016).

Figure 3C illustrates the T secretion rate in the control and study groups. In all groups, the T secretion rate was not significantly altered throughout the culture period. The mean peak T level was of 0.12 ± 0.05 in the control group, 0.31 ± 0.26 ng/ml in the sema-4D group, and 0.12 ± 0.01 ng/ml in the group treated with neutralizing antibodies against Plexin-B1.

## Discussion

Immunohistochemical studies revealed staining for Sema-4D in the mouse ovary at all stages, mainly in the peri-luminal granulosa cells. The staining became more evident following PMSG or hCG injection. Following hCG injection, Sema-4D was observed in the lumen. Sema-4D is the only semaphorin for which membrane and soluble forms are endowed with functional properties, and for which bidirectional signaling has been suggested. Both human and mouse Sema 4D are cleaved and released from the membrane as a 120 kDa soluble form [13]. Sema-4D transcripts are observed at high levels in both lymphoid and non-lymphoid organs including the brain, heart, kidney, spleen, thymus, and lymph nodes [14]. The increase in Sema-4D staining after PMSG and hCG might suggest that it is under hormonal regulation. However, we cannot exclude the possibility that as a result of hormonal regulation, other changes that occur within the follicle, i.e. growth factors secretion, might be involved in Semaphorin-4D expression. In our previous study we found similar patterns of expression for Plexin B1 in the mouse ovary [15]. The finding that Plexin-B1 ligand, Sema 4D, is also expressed in the mouse follicle and increases after hormonal treatment suggests follicular auto-regulation of the Plexin/Semaphorin system.

Since there is no known Semaphorin inhibitor, the effect of the ligand had to be studied by stimulation. In this study, the addition of Sema-4D revealed no significant difference in follicular diameter. However, the time needed to reach maximal diameter in the Sema-4D treated group was significantly shorter.

In this study we could not examine endothelial cell-formation, however one can speculate that the growing follicle require induction of angiogenesis together with growth and remodeling of new blood vessels from a pre-existing vascular network, to ensure the delivery of oxygen, nutrients, and growth factors, similar to tumor growth [7]. It is also well documented that Sema-4D, along with its receptor, Plexin-B1, participate in tumor growth and proliferation [16]. Semaphorins, such as Sema-4D, can generate local membrane signals that more finely regulate cell motility [9]. Moreover, through their action on cytoskeleton, Semaphorins can induce cytoskeletal re-arrangements only in those portions of the target cells facing surrounding cells expressing plexin on their membranes. This could allow a finer tuning of directional movements [17]. Moreover, interaction between these two families of receptors unveils a new mechanism that could play a role in the process of follicular growth [18].

In the control group, after the addition of hCG to the culture, follicular diameter was reduced. This could be explained by rapid follicular rupture which mimics the follicular rupture that occurs following ovulation (i.e. granulosa collapse into the lumen). It is known that the addition of hCG causes a change in the follicular phase from growing phase into differentiating phase by causing granulosa cell luteinization [19]. At this stage granulosa cells lose their proliferating capacity. The addition of neutralizing antibodies against Plexin-B1 to the Sema-4D treated group abolished this stimulatory effect on follicular growth. These antibodies are directed against the ligand-binding domain on the receptor, inhibiting Plexin-B1 activity by competing with Sema-4D, the natural ligand. This finding further supports our hypothesis that Semaphorin 4D acts via its receptor, plexin B1.

In our previous study we have shown that the addition of neutralizing antibodies against Plexin-B1 causes a significant reduction in follicular growth. In the present study, the addition of Sema-4D increased steroid hormone secretion rate when compared with the control group, and neutralizing antibodies against Plexin-B1 abolished this effect. Both estrogen and progesterone secretion patterns were different between the control and the Sema-4D treated groups. While estradiol constantly increased, and remained high in the control group, in the Sema-4D group estradiol level reached its peak and was subsequently reduced to a lower secretion level. The reverse pattern was observed in progesterone secretion. In the control group, one day after hCG injection, a reduction in progesterone secretion was demonstrated, while in the Sema-4D treated group progesterone secretion rate reached its peak only three days after the addition of hCG.

The signaling pathways mediated by the Plexin/Semaphorin system have not been fully elucidated. We cannot exclude the possibility that changes in cytoskeleton re-organization influence receptor expression, or that stimulating the Plexin/Semaphorin-cascade influences down stream cytoplasmic signals involved in steroidogenesis. It has been reported that the cytoplasmic domain of plexin-B1 associates with the activated form of the small GTPase R-Ras, and promotes its inactivation through an intrinsic R-Ras-GAP activity [20]. Binding of Semaphorin to Plexin initiates a signaling cascade, which varies depending on the Plexin/Semaphorin/family pair. The down stream cascade involves the activation of MAPK, which is also known to be involved in steroidogenesis. It should be stressed that since Sema-4D already exists in the developing follicle, one cannot exclude the possibility that the addition of Sema-4D creates a supra-physiologic effect.

Nevertheless, these results suggest that Sema-4D modulates follicular development rates. The mechanism by which Sema-4D/Plexin-B1 system affects follicular growth and steroidogenesis demands clarification.

## Authors' contributions

AR took part in designing the study and analyzing the data, performed the experiments and drafted the manuscript. SG took part in designing the study and analyzing the data, controlled all the experiments, and revised all drafts of the manuscript. ES designed the study, analyzed the data and edited the drafts of the manuscript. All authors read and approved the final manuscript.
